# P31 WHO GLASS participation and antimicrobial resistance in 55 South Centre Member States: gaps and stewardship insights

**DOI:** 10.1093/jacamr/dlag102.037

**Published:** 2026-06-26

**Authors:** Rasha Abdelsalam Elshenawy

**Affiliations:** School of Health, Medicine and Life Sciences, University of Hertfordshire, Hatfield, UK

## Abstract

**Background:**

Robust antimicrobial resistance (AMR) surveillance is foundational to effective stewardship and global health security. The WHO Global Antimicrobial Resistance and Use Surveillance System (GLASS), established in 2015, provides the primary international framework for standardized monitoring of AMR and antimicrobial use (AMU) [1]. Despite its expansion to 109 countries by 2023, significant underrepresentation of low- and middle-income countries (LMICs) persists, limiting the global visibility of resistance trends in regions bearing the highest burden [2].

**Objectives:**

To evaluate the participation of the 55 South Centre Member States, representing developing countries across Africa, Asia, Latin America, and the Caribbean, in WHO GLASS AMR and AMU surveillance from 2016 to 2023, and to identify patterns, gaps, and determinants of engagement.

**Methods:**

A cross-sectional descriptive study design was employed. Data were extracted from the publicly available WHO GLASS digital dashboard on 18 November 2024. Country-level enrolment status, year of registration, and participation in AMR and AMU surveillance modules were systematically recorded for all 55 South Centre Member States. Findings were cross-verified against WHO GLASS Annual Reports 2022–2024. Descriptive statistics characterized enrolment patterns by region, time period, and surveillance type. Pre-pandemic (2016–2019) and pandemic-era (2020–2021) enrolment rates were compared. Country case studies (India, South Africa) were analysed to contextualize data quality and representativeness.

**Results:**

By end of 2021, 33 of 55 South Centre Member States (60%) had enrolled in GLASS-AMR surveillance, rising from 7 countries in 2016 to a peak of 8 new enrolments in 2017. The COVID-19 pandemic substantially disrupted engagement, with new enrolments declining by 77% during 2020–2021 compared to the pre-pandemic period. AMU surveillance participation was markedly lower: only 20 countries (38%) had enrolled by end of 2023, with just 15 countries (27%) participating in both components. African nations constituted the largest regional group (45%), followed by Asia (33%) and Latin America and the Caribbean (21%). Country case studies demonstrated that sustained GLASS participation directly enables targeted AMS: India's surveillance revealed *Escherichia. coli* resistance exceeding 80% to third-generation cephalosporins, informing empirical prescribing guidelines, while South Africa achieved 84.1% Access antibiotic usage, exceeding the WHO 60% GPW13 target. Structural gaps across member states included inadequate laboratory capacity, fragmented pharmaceutical data systems, limited digital infrastructure, rural exclusion, absent One Health integration, and workforce shortages.

**Conclusions:**

Despite meaningful progress, critical gaps remain in AMR and AMU surveillance across 55 South Centre Member States, undermining evidence-based stewardship. Country-specific GLASS data provides a clear epidemiological picture enabling tailored AMS interventions, replacing generic guidelines with context-appropriate responses. Robust surveillance is equally fundamental to emergency preparedness, as the pandemic demonstrated. Closing data gaps through integrated national AMR action plans, interoperable digital infrastructure, and regional capacity building is both a stewardship and health security imperative for equitable, targeted global antimicrobial stewardship.
